# Can optical spectral transmission assess ultrasound-detected synovitis in hand osteoarthritis?

**DOI:** 10.1371/journal.pone.0209761

**Published:** 2019-02-22

**Authors:** N. J. Besselink, J. W. G. Jacobs, A. A. A. Westgeest, P. van der Meijde, P. M. J. Welsing, A. C. A. Marijnissen, F. P. J. G. Lafeber, W. E. Van Spil

**Affiliations:** 1 Department of Rheumatology & Clinical Immunology, University Medical Center Utrecht, Utrecht, The Netherlands; 2 Department of Rheumatology, Máxima Medisch Centrum, Eindhoven, The Netherlands; 3 Department of Rheumatology, Noordwest Ziekenhuisgroep, Alkmaar, The Netherlands; Monash University, AUSTRALIA

## Abstract

**Objective:**

To determine whether optical spectral transmission (OST) can be used to assess synovitis in hand and wrist joints of patients with hand osteoarthritis (OA).

**Design:**

Hand and wrist joints of 47 primary hand OA patients with at least one clinically inflamed hand or wrist joint were assessed for synovitis by OST and ultrasound (US). Associations between standardized OST and US synovitis were studied in linear mixed effects models, across all joint types together and individually for wrist, proximal interphalangeal (PIP), and distal interphalangeal (DIP) joints, and were adjusted for OA features that showed associations with US synovitis. Diagnostic performance was determined using receiver operator characteristic (ROC) curves analysis, with US as reference standard.

**Results:**

Altogether, 6.7% of joints showed US synovitis. Statistically significant associations between OST scores and US synovitis were found for all joints combined (Δ0.37SD, p<0.001) and PIP joints (Δ0.81SD, p<0.001), but not for DIP (Δ0.14SD, p = 0.484) or wrist joints (Δ0.37SD, p = 0.178). All associations were independent of other OA features, i.e. osteophytes and dorsal vascularity. Analysis of diagnostic performance of OST, revealed an area under the ROC curve (AUC-ROC) of 0.74 for all joints together (p<0.001), 0.69 for PIP joints (p<0.001), 0.54 for DIP joints (p = 0.486), and 0.61 for wrist joints (p = 0.234).

**Conclusions:**

OST scores and US synovitis are statistically significantly associated, independent of osteophytes and dorsal vascularity. At this stage, OST performs fair in the assessment of synovitis in PIP joints of hand OA patients.

## Introduction

Hand osteoarthritis (OA) is a highly prevalent, multifactorial joint disease that poses a huge and ever growing burden to affected individuals and society. Synovitis can occur at any disease stage and is an acknowledged risk factor for OA progression [1], probably through pro-inflammatory mediators that affect the joint tissues [2]. Synovitis in hand joints with OA adds to pain, functional impairment, and progression of joint damage [3–5]. The subgroup of hand OA patients with so-called inflammatory or erosive hand OA is characterized by more rapid disease progression, by more pain, by functional impairment and inflammatory symptoms and signs, and by more negative clinical, laboratory and sonographic outcomes as compared to other hand OA patients [6]. Early assessment and treatment of synovitis in hand OA patients might provide a potential opportunity to delay or even prevent joint deterioration.

Various imaging methods are used to assess signs of synovitis, such as magnetic resonance imaging (MRI) or ultrasound (US). While the sensitivity of MRI and US in detecting synovitis and tenosynovitis is higher than that of clinical examination. MRI and US can be rather time-consuming, observer-dependent, and/or costly. Therefore, alternative methods for objective and fast assessment of synovitis are desired. For example, high intensities in indocyanine green (ICG)-enhanced fluorescence optical imaging are suggestive of synovitis in wrists and proximal interphalangeal (PIP) joints of primary hand OA patients [7].

The novel optical spectral transmission (OST) technique might be an attractive alternative to US and MRI. OST is able to objectively measure the reduction of light transmitted through joint tissues in presence of inflammation (e.g. synovitis, tenosynovitis). A recent study showed that OST had a sensitivity of 60% and specificity of 89% for assessing US synovitis in hand and wrist joints of rheumatoid arthritis (RA) patients, even in the presence of bone pathology and periarticular tendon inflammation [8]. Also, OST correlated stronger with US signs of synovitis (ρ = 0.64, 95%-CI: -0.43 to 0.78, p<0.01) than swollen joint count (SJC: ρ = 0.30, 95%-CI: 0.11 to 0.46, p<0.01) and the tender joint count (TJC: ρ = -0.02, 95%-CI: -0.21 to 0.17, p = 0.84).

The current study aims to assess performance of OST in assessing synovitis in hand and wrist joints of patients with hand OA and clinical signs of synovitis in at least one hand or wrist joint. Hitherto, we first, determine the test-retest reliability of OST measurements. We then compare OST levels between joints with and without US synovitis, adjusting for relevant other OA features. Finally, we determine diagnostic performance from receiver operator characteristic (ROC) curves, using US synovitis as a reference.

## Methods

### Subjects

Fifty consecutive outpatients with primary hand OA were recruited at the outpatient clinic of the Department of Rheumatology & Clinical Immunology at the University Medical Center Utrecht (UMC Utrecht), between October 2016 and March 2017. Patients were eligible when they had at least one swollen finger or wrist joint (by clinical examination), were aged over 18 years, and were able to give informed consent. Exclusion criteria were obvious wrist or hand deformations, hand or wrist joint prostheses, inflammatory rheumatic diseases that can affect hand or wrist joints (e.g. RA, psoriatic synovitis, gout), intra-articular glucocorticoid injections of hand or wrist joints within the past 3 months, trauma or surgery of hand or wrist joints within the past 6 months, light hypersensitivity (e.g. due to erythropoietic protoporphyria), and/or photodynamic therapy in the past or near future. For three patients, data could not be used because of movement artefacts or incorrect positioning during scanning, leaving data of 47 patients for analysis.

### Assessment procedure

Experienced examiners, blinded for other study results, performed OST and US assessments. The study complied with the Declaration of Helsinki. All patients gave written informed consent. The ethics committee of the UMC Utrecht (*NL 50848*.*041*.*15*) approved the study protocol.

### Optical spectral transmission

**Fig 1:**
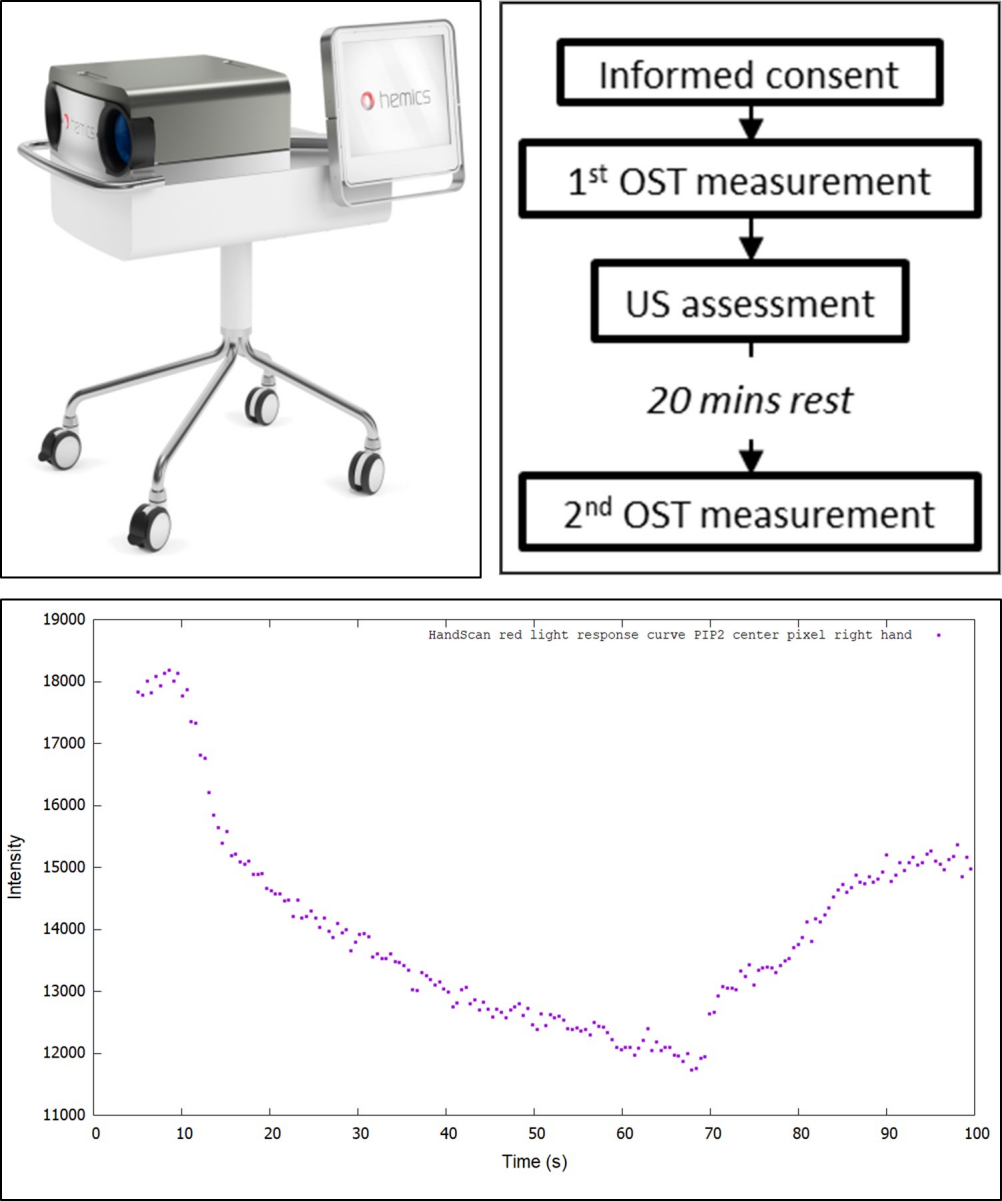
A) The HandScan apparatus. B) Data on the hemodynamic response to venous occlusion that are derived from light transmission data (described in more detail previously [9]). C) The study procedure for US and OST assessment.

OST measurements were performed by one experienced nurse using the HandScan (Hemics BV., Eindhoven, The Netherlands). For an OST measurement, the hands are inserted through cylindrical openings that contain pressure cuffs (Fig 1A). In less than 100 seconds, light transmission data is obtained and transformed into blood flow parameters for each hand and wrist joint (Fig 1B). Previously created RA algorithms to determine inflammation by OST could not be applied to the current hand OA cohort as these diseases differ in commonly affected joints, severity of synovitis, and presence of osteophytes [9]. Therefore, image analysis, algorithm development, and algorithm validation were specifically performed for the current OA cohort. Development and validation of the algorithm were performed as was previously described in detail for the RA patients,[8] this time also including the distal interphalangeal (DIP) joints and carpometacarpal (CMC)-1 joints.

The algorithm provides an OST index for each joint (CMC1, DIP 2–5, (P)IP1–5, metacarpophalangeal (MCP) 1–5, and the wrists of both hands; range: 0–3), and a total OST index, being the average of all joints, scaled to a total OST index range of 0–66, to make comparison to total OST index for RA possible [8]. To determine the test-retest reliability, duplicate OST assessments were performed. OST assessment was performed before and after US, with at least 20 minutes of rest after US examination allowed for normalization of blood flow, as illustrated in Fig 1C.

### Ultrasonography

US examination was performed by a single experienced examiner (PvdM) using a MyLab 60 system (Esaote, Genua, Italy) with an 18–6 MHz linear array transducer. Patient and probe positioning were performed according to EULAR guidelines [10]. Dedicated scoring systems for US synovitis in hand OA are not yet available. We, therefore, used the standardized scoring system from the Outcome Measurements in Rheumatology Clinical Trials (OMERACT) guidelines that is intended to assess synovitis in hand and wrist joints of RA patients [11]. In this scoring system, greyscale US (GSUS) findings on joint effusion and synovial thickening [12], as well as power Doppler (PDUS) findings are graded on a semiquantitive scale ranging between 0 and3 [13,14]. Features that might potentially lead to misclassification of synovitis by OST were also assessed: extensor tendinitis, flexor tenosynovitis, dorsal vascularity, osteophytes, and erosions. Assessment of synovitis, tendinitis/tenosynovitis, and features potentially misclassifying synovitis, were described in more detail previously.[8]

With GSUS grade 1 findings frequently present in health controls,[15] US joint inflammation was defined as GSUS synovitis >1 or PDUS synovitis >0 or a GSUS/PDUS tenosynovitis score >0.

### Statistical analysis

Associations between the presence of US synovitis and standardized OST score were assessed for all joints combined and for joint groups individually. Individual joint groups were only studied when US synovitis was present in >5% of joints, to increase the likelihood of sufficient relevance and statistical power. As explained before, associations between US and OST were adjusted for potential US confounders. Due to limited numbers of subjects and US findings, a preselection of potentially relevant confounders was performed: potential confounders were selected when Chi-Square tests for the association between the presence of US synovitis and the potential confounder showed p-values <0.1.

The association between US synovitis and OST values in individual joints was studied using multilevel analysis (i.e. a linear mixed effects model) to account for the dependence of measurements within patients and between sides (left or right). Normalized OST values were used as the dependent (outcome) variable (i.e. raw OST values transformed into z-scores having a mean of zero and a standard deviation (SD) of one). The consequences of having US synovitis, joint type, side (left or right), and presence of potentially confounding US features on normalized OST values were evaluated as fixed effects in the model. Random intercepts at the level of patient, joint type and side (left, right) were evaluated in all analyses and retained when they improved model fit (i.e. lower Bayesian information criterion values). The regression coefficients that are derived from these analyses can be interpreted as the increase in OST value (in SD units) in joints with US synovitis compared to joints without US synovitis. The impact of joint type, side and potential confounders on OST value and the association of US synovitis and OST will also be evaluated in this model.

### Diagnostic performance of OST

Test-retest reliability of OST was evaluated by intra-class correlation coefficients (ICC), and Bland-Altman plots, at both joint (individual joint OST) and patient level (total OST).

Diagnostic performance of OST was determined using US as a reference (scoring synovitis as absent or present), by receiver operating characteristic (ROC) curve analyses with 95% confidence interval (95%-CI) estimation. This was also done for individual joint groups when more than 5% of the joints of that group showed US synovitis.

Multiple regression analyses to develop the OST algorithm were performed using proprietary third party software by Hemics (InFlame RA-160205, November 3, 2016). All other analyses were performed by using SPSS (IBM Corp. Released 2012. IBM SPSS Statistics for Windows, Version 21.0. Armonk, NY: IBM Corp.). All tests were two-sided; p-values < 0.05 were considered statistically significant.

## Results

Patient demographics are reported in Table 1; 89% was female, and the average age was 64 years. No adverse events occurred.

**Table 1:** Patient demographics and imaging data.

n = 47	
**Age (years)**	64.5 (9.9)
**Female (N, %)**	42 (89%)
Total US inflamed joints (N, 0–32)*	2 (IQR: 1–3, range 0–7)
**Total OST score (0–66)**^$^	9.27 (0.83)

Data are presented as mean (SD) or median (IQR, range) unless mentioned otherwise. US synovitis was defined as GSUS synovitis >1 or PDUS synovitis >0.

* Total number of inflamed joints per patient as assessed by US.

^$^Total OST score: the average of all joints times 22, to maintain a similar total OST index as for the previous RA cohort. [8] US, ultrasound; OST, optical spectral transmission.

### US observations

As illustrated in Table 2, 6.7% of all joints were inflamed according to US. Joint types of interest for evaluation at joint group level, exceeding the arbitrary cut-off of synovitis in >5%, were the wrist (N = 12, 12.8%), PIP (N = 55, 11.7%), and DIP (N = 23, 6.1%) joints. The prevalence of potential confounders varied, erosions were absent at the dorsal side and very rare at the volar side (4/1400). Tendinitis was also rare, with more flexor (1.1%) than extensor (0.2%) tendinitis. Dorsal vascularity was observed quite often (17.7%) and osteophytes were present in 44.3% of all joints. Only dorsal vascularity and osteophytes were related to presence of US synovitis (i.e. Chi-square test P < 0.1) and, therefore, included as potential confounders in further analyses, see Table 3.

**Table 2:** Descriptives for all joints together and for each separate joint group.

	N (US-synovitis)	US GS grades				US PD grades				Erosions scanned dorsally	Erosions scanned volarly	Flexor tendinitis	Extensor tendinitis	Dorsal vascular pattern	Osteophytes
		*0*	*1*	*2*	*3*	*0*	*1*	*2*	*3*						
All joints	100/1503 = 6.7%	1403	31	64	5	1435	50	16	2	0/1314 = 0%	4/1400 = 0%	16/1307 = 1.1%	3/1312 = 0.2%	266/1313 = 17.7%	666/1314 = 44.3%
DIP 2–5	**23/375 =** **6.1%**	352	9	14	0	362	12	1	0	0/375 = 0%	2/370 = 0.5%	5/370 = 1.3%	0/375 = 0.0%	142/375 = 37.9%	294/375 = 78.4%
PIP 1–5	**55/470 =** **11.7%**	415	14	38	3	431	25	13	1	0/469 = 0%	2/467 = 0.2%	6/467 = 1.3%	2/468 = 0.4%	121/468 = 25.7%	317/469 = 67.4%
CMC 1	2/94 = 2.1%	92	0	2	0	92	1	1	0	NA	0/93 = 0%	NA	NA	NA	NA
MCP 1–5	8/470 = 1.7%	462	2	6	0	465	4	1	0	0/470 = 0%	0/470 = 0%	5/470 = 1.1%	1/470 = 0.2%	3/470 = 0.6%	55/470 = 11.7%
Wrist	**12/94 =** **12.8%**	82	6	4	2	85	8	0	1	NA	NA	NA	NA	NA	NA

An arbitrary cut-off for analysis of OST performance in individual joint types was set at 5% affected joints, to focus on the most relevant joint groups and maintain sufficient power, leaving DIP, PIP and wrist joints. GS: grayscale US scores (0–3), PD: power Doppler US scores (0–3).

**Table 3:** Cross-tabulation of US synovitis and other, potentially confounding, US variables.

		Erosions scanned dorsally		Total	Erosions scanned volarly		Total	Flexor tendinitis		Total	Extensor tendinitis		Total	Dorsal vascular pattern		Total	Osteophytes		Total
		0	1		0	1		0	1		0	1		0	1		0	1	
US arthritis	0	1228	0	1228	1309	3	1312	1206	15	1221	1223	3	1226	985	242	1227	638	590	1228
	1	86	0	86	87	1	88	85	1	86	86	0	86	62	24	86	10	76	86
Total		1314	0	1314	1396	4	1400	1291	16	1307	1309	3	1312	1047	266	1313	648	666	1314
Pearson Chi-Square:		-		-	2.385		p = 0.123	0.003		p = 0.957	0.211		p = 0.646	3.332		**p = 0.068**	52.291		**p<0.001**

Chi-Square tests were used to determine whether a potentially confounding US variable was related to the distribution of US synovitis. If the p-value was <0.1, that variable was tested as a potential confounder of the association between OST values and US synovitis in further analyses.

### Test-retest reliability of OST

**Fig 2:**
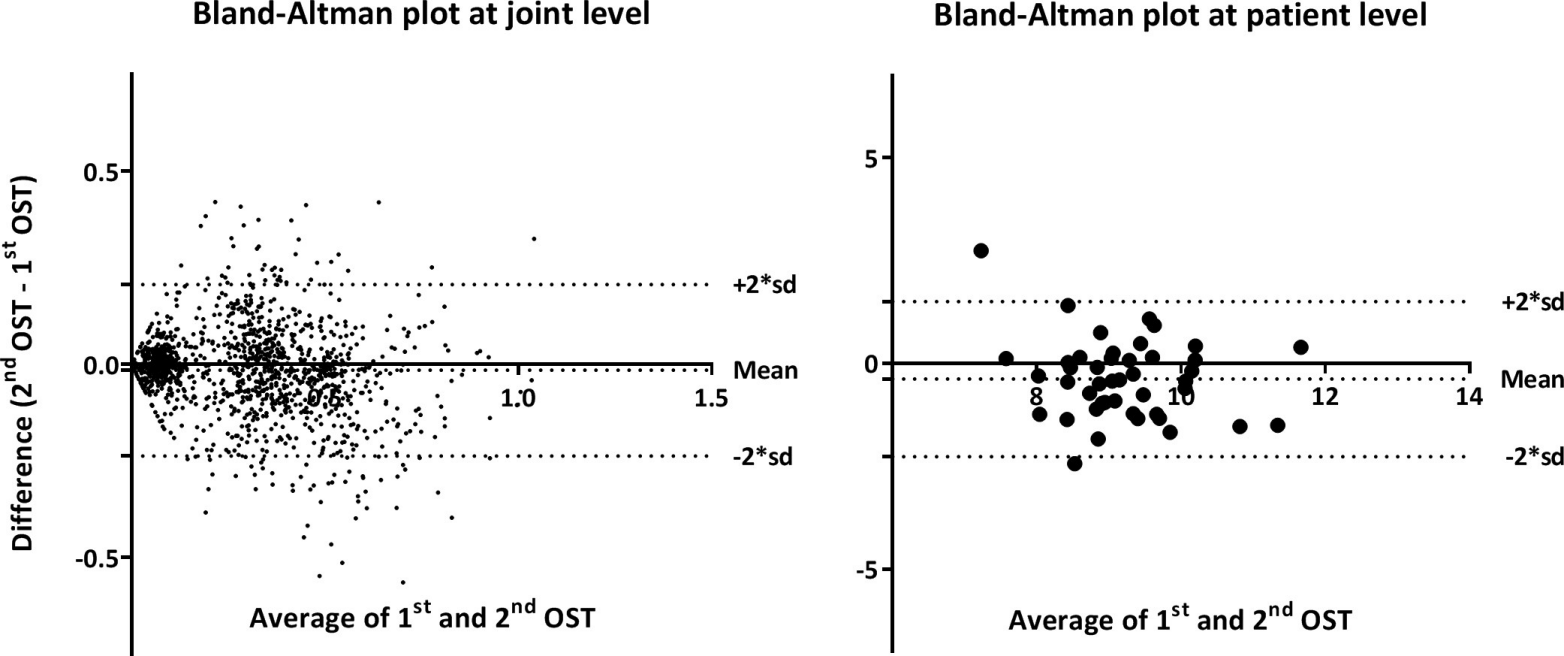
Test-retest agreement of OST. Bland-Altman plots of duplicate OST measurements at patient level (OST index range: 0–66) and at joint level (OST index range: 0–3). 1^st^ OST: first OST measurement, 2^nd^ OST: second OST measurement.

As illustrated in Fig 2, repeated OST measurements (with US assessment and 20 minutes of rest in between) were essentially similar to the initial measurements at both joint level (mean difference: 0.01, 95%-CI: -0.237 to 0.207, n.s.) and patient level (mean difference: -0.38, 95%-CI: -2.30 to 1.50). ICC for two-way mixed single measurement (ICC 3,1) showed excellent reliability at joint level (ICC = 0.82, 95%-CI: 0.80 to 0.83, p<0.001) and fair reliability at patient level (ICC = 0.54, 95%-CI: 0.29 to 0.72, p = 0.001).

### Associations between OST and presence of US synovitis

In the final model (Table 4), a statistically significant association of the presence of US synovitis with higher OST values was found (0.37 SD units, p<0.001). OST values were also different between joint groups (p<0.001) and sides (right vs. left: Δ0.05 SD p = 0.047). As joint group appeared to modify the association between US synovitis and OST values (p<0.001 for interaction), stratified analyses per joint group (wrist, PIP, and DIP joints) were performed. Table 4 shows a strong association between US synovitis and OST in PIP joints (Δ0.81 SD, 95%-CI: 0.56 to 1.06, p<0.001), but not in DIP (Δ0.14 SD, 95%-CI: -0.26 to 0.55, p = 0.484) or wrist joints (Δ0.37 SD, 95%-CI: -0.17 to 0.91, p = 0.178).

**Table 4:** Results of multilevel (mixed effects) linear regression model with OST values as the dependent outcome: Association with US synovitis, for all joints combined and for individual joint types.

Parameter	Estimate^*$*^	95% Confidence Interval		Sig.
		Lower Bound	Upper Bound	
**Overall model**				
*Presence of US synovitis*	0.37	0.28	0.46	**P<0.001**
*PIP vs*. *wrist*	0.68	0.55	0.82	**P<0.001**
*DIP vs*. *wrist*	-0.20	-0.33	-0.06	**P = 0.005**
*MCP vs*. *wrist*	-1.34	-1.48	-1.21	**P<0.001**
*CMC vs*. *wrist*	-1.44	-1.59	-1.29	**P<0.001**
*Left vs*. *right side*	-0.05	-0.09	0.00	**P = 0.047**
**Effect of presence of US synovitis for individual joint groups**				
*Wrist*^$^	0.37	-0.17	0.91	P = 0.178
*PIP*^$^	0.81	0.56	1.06	**P<0.001**
*DIP*^$^	0.14	-0.26	0.55	P = 0.484

^$^ Estimates indicate the number of SD that OST values (dependent variable) change from one unit increase in the listed parameters (independent variables). The association between OST values and US synovitis in individual joints was studied using multilevel analysis. Joint group appeared to modify the association between US synovitis and OST values (p<0.001 for interaction) and, therefore, associations between OST and US synovitis are shown for each joint group as well.

P-values <0.05 are written in bold.

The identified potential US confounders osteophytes and dorsal vascularity were only available for PIP and DIP joints. In these two joint types combined, it appeared that osteophytes (p = 0.603) and dorsal vascularity (p = 0.813) had no statistically significant association with OST values. Moreover, no modification of the association between US synovitis and OST by osteophytes (p = 0.267 for interaction term) and dorsal vascularity (p = 0.409 for interaction term) was established.

### Diagnostic performance of OST

For all OST assessments combined, data were available for 1503 joints (16 joints per hand in 47 patients, minus one joint that was excluded due to a ring that could not be removed). From these data an algorithm was developed to assess US synovitis in this cohort. Details on algorithm development and validation have been discussed in detail previously.[8] The limited number of inflamed joints and the overall mild synovitis in this cohort made algorithm development considerably difficult, particularly for the DIP joints.

**Fig 3:**
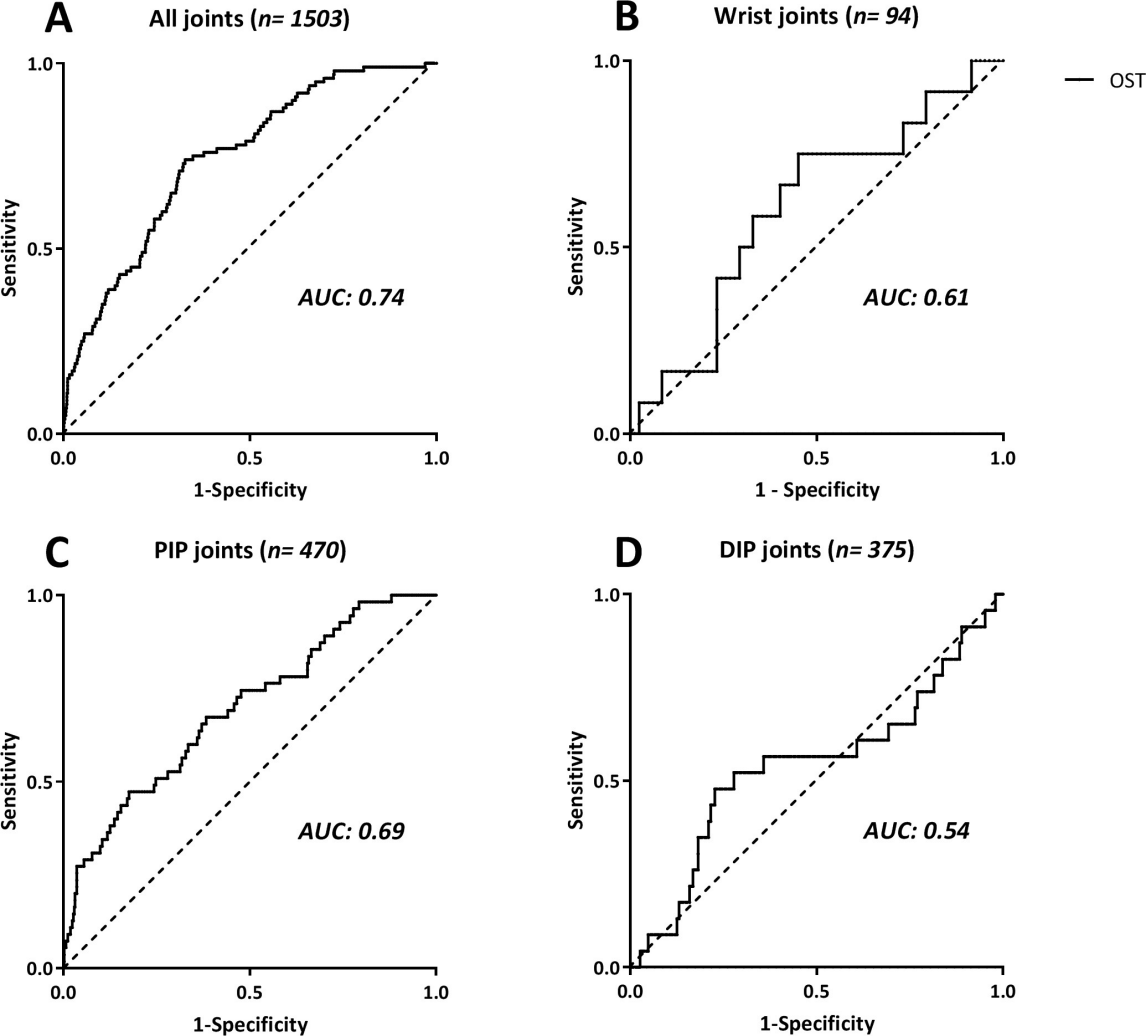
Receiver Operating Characteristic (ROC) curves. Areas under the curves (AUC) for optical spectral transmission (OST) in demonstrating ultrasonography observed synovitis (US synovitis) in A) all joints together (AUC-ROC: 0.74, 95%-CI: 0.70 to 0.79, p<0.001), B) wrist joints (AUC-ROC: 0.61, 95%-CI: 0.44 to 0.77, p = 0.234), C) PIP joints (AUC-ROC: 0.69, 95%-CI: 0.62 to 0.77, p<0.001), and D) DIP joints (AUC-ROC: 0.54, 95%-CI: 0.41 to 0.68, p = 0.486).

Accordingly, as shown in Fig 3, OST performance for all joints together (AUC-ROC: 0.74, 95%-CI: 0.70 to 0.79, p<0.001) was higher than for the DIP (AUC-ROC: 0.54, 95%-CI: 0.41 to 0.68, p = 0.486) and the wrist joints (AUC-ROC: 0.61, 95%-CI: 0.44 to 0.77, p = 0.234), but similar to the (P)IP 1–5 joints (AUC-ROC: 0.69, 95%-CI: 0.62 to 0.77, p<0.001).

## Discussion

The current study demonstrates the diagnostic performance of OST for detecting US defined synovitis in patients with inflammatory hand OA. OST values are statistically significantly higher in the presence of US-defined synovitis, independent of the concurrent presence of osteophytes and dorsal vascularity. This association was strongest when all wrist and hand joints were combined and for PIP joints in particular.

Diagnostic performance of OST was fair for all joints combined and for PIP joints, but poor for the wrist and DIP joints. US synovitis was relatively uncommon in our cohort, even though patients were required to have at least one clinically swollen hand or wrist joint. There were 11 patients without any synovitis in hand or wrist joints according to US (i.e. GS>1 | PD>0). The low number of inflamed joints and low intensity of synovitis, although maybe typical for an OA cohort, posed difficulties for setting up an effective diagnostic algorithm for other joint groups than the PIP joints. The lesser performance of OST for the wrist could be due to the more complex anatomy of the wrist as compared to the PIP and DIP joints, and is in line with our previous findings in RA patients. [8] While synovitis in DIP joints is relatively common in hand OA, they might be of less clinical relevance, as they supposedly cause less functional limitations than inflamed PIP joints do. It is difficult to put the current findings in the light of existing literature, as previous research on OST focused on RA patients with more inflamed joints and more severe synovitis [8,16,17], used a different light-source,[16,17] assessed different joints, and/or used clinical examination as a reference standard.[17]

Previous studies in hand OA patients, suggest a dose-response association between US synovitis and radiographic OA progression. The risk of OA progression in joints with GSUS grade 1 is higher compared to that of joints without GSUS signal [18]. However, US also has its limitations for assessing synovitis. First, GSUS≠0 and GSUS = 1 in particular can also be found in healthy individuals [15]. Defining for synovitis as GSUS grade ≥ 1, irrespective of PDUS grade, would have yielded 263 more arthritic joints in our study. When this alternative definition was used in the ROC curve analysis, diagnostic performance of OST appeared to be almost similar though (ROC-AUC: 0.737 to 0.722, p = 0.654). Second, in a study comparing US and MRI assessment of synovitis in MCP and PIP joints of RA patients, US was demonstrated to have a sensitivity and specificity between 0.6 and 0.8 as compared to MRI. Importantly, US appeared to perform less in joints with subclinical synovitis in particular, as might have been more prevalent in our current OA cohort [19]. Likewise, another study on the performance of OST in RA also showed a moderate correlation between OST and MRI synovitis (r = 0.52, p = 0.005) in RA patients with low disease activity [16]. Therefore, OST performance might be underappreciated in the current study, and in turn, performance of the reference standard might be reduced because of the implemented scanning and scoring guidelines that were limited to predefined areas.

This study is the first to evaluate OST for the assessment of synovitis in hand OA, but has some limitations. First, US was performed by a single, though very experienced, examiner. Inter-observer reliability of the US assessment could therefore not be determined. However, the reliability US for detecting synovitis in hands and wrists joints, using the same semiquantitative scale, is considered to be high in general [12,20]. Nevertheless, in future studies it would be preferable to perform duplicate US assessment, to be able to correct for measurement errors. Second, the number of joints with synovitis and the severity of the synovitis in this study were low. This caused suboptimal development of the OST algorithm. In our study, out of the 1536 joints that could be successfully assessed by US, there was an uneven distribution of inflammation grades among all joints (S1 Fig). Moreover, PIP joints showed more severe US joint indices (US joint index >2). Although explicit data on the distribution and severity synovitis among wrist and hand joints of hand OA patients are scarce, similar distributions were found in other studies. This increases the chance of successful validation of our model in another cohort of hand OA patients without obvious deformations. Third, the algorithm was developed and validated in the same cohort. Yet, several precautions were taken to prevent overfitting of the OST algorithm, such as increasing the dataset (duplicate measurements), using leave-one-out cross validation, and parameter reduction (described in more detail previously [9]). Fourth, in the future, improved OST algorithm should be compared to physical examination, and other reference standards like MRI. Fifth, although fast and objective assessment of synovitis is an obvious advantage of OST, using alternative imaging techniques like MRI, would have the advantage of also being able to assess subchondral bone, cartilage, and fat tissue.

In summary, OST values are statistically significantly higher in the presence of US synovitis. This overall association between OST values and US synovitis relies on strong associations at the level of PIP joints though and less so in the associations at the level of the wrist and DIP joints. These results seem promising. Future studies, preferably in cohorts of hand OA patients showing more severe synovitis and also including MRI data on synovitis, to study the added value of OST in clinical practice and research would be of great interest and are eagerly awaited.

## Supporting information

S1 Fig Percentage of joints per US Joint Index* per joint type. *The sum of the maximum of GSUS and PDUS grades for synovitis. Distribution of joints per US joint index, for all joints pooled together, and separately for the DIP, PIP, and wrist joints. US joint index range: 0–6.
(TIF)
