# Supplementary figures and images for: Can optical spectral transmission assess ultrasound-detected synovitis in hand osteoarthritis?
Source: PLoS One. 2019 Feb 22;14(2):e0209761. doi: 10.1371/journal.pone.0209761 (PMC6386475; doi:10.1371/journal.pone.0209761)

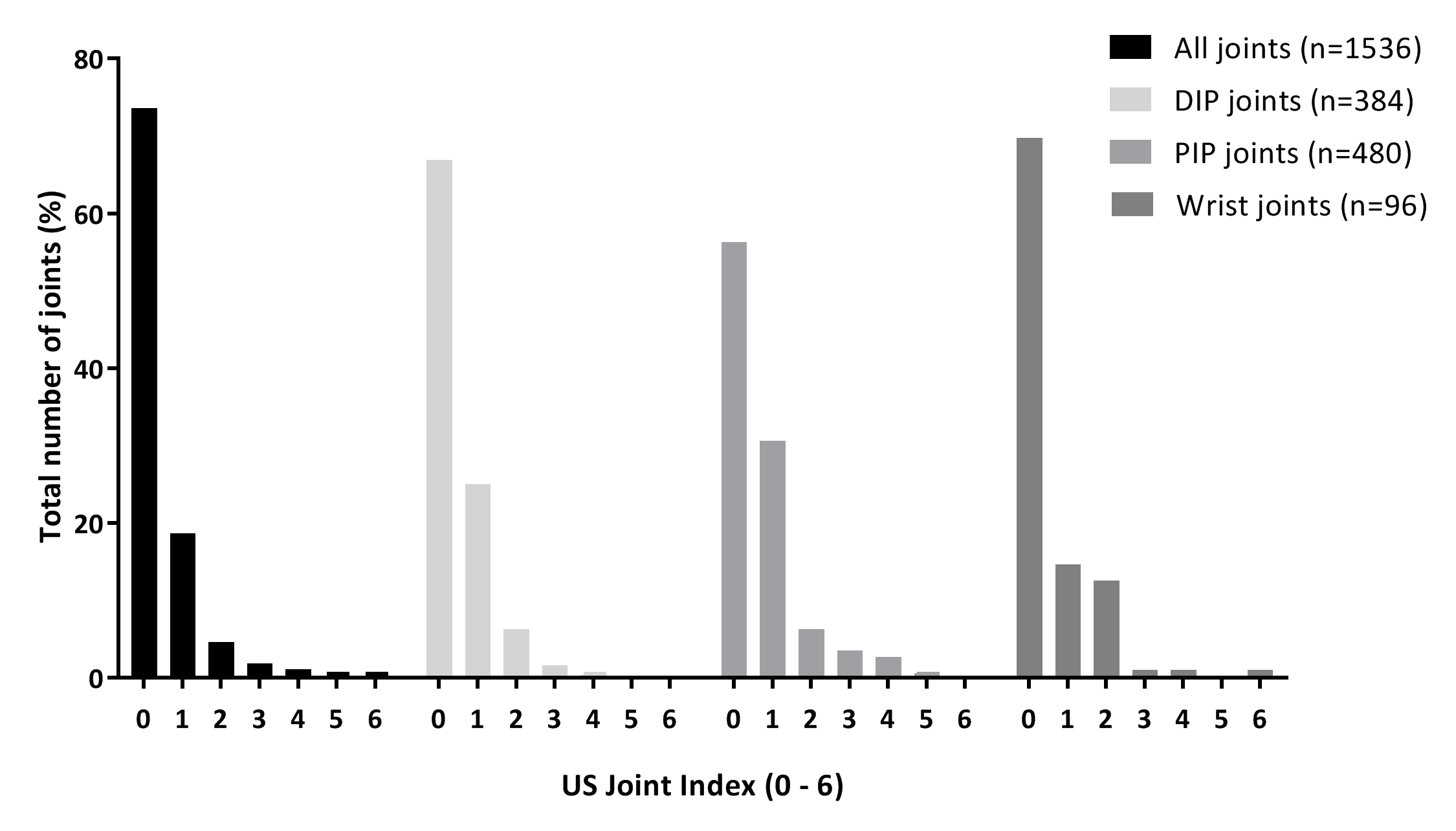

Supplement: S1 Fig — *The sum of the maximum of GSUS and PDUS grades for synovitis. Distribution of joints per US joint index, for all joints pooled together, and separately for the DIP, PIP, and wrist joints. US joint index range: 0–6. (TIF) [file pone.0209761.s001.tif]
